# A biopsychosocial approach to parents of children with juvenile idiopathic arthritis

**DOI:** 10.1186/1546-0096-9-S1-P115

**Published:** 2011-09-14

**Authors:** Z Georgiadou, M Trachana, P Pratsidou-Gertsi, G Pardalos, M Stavrakidou, I Karagiannaki

**Affiliations:** 1 Department of Child Psychiatry, Ippokration General Hospital, Thessaloniki, Greece; 2 Pediatric Immunology and Rheumatology Referral Center, First Dept of Pediatrics, Aristotle University, Thessaloniki, Greece

## Background

The burden of Juvenile Idiopathic Arthritis (JIA) in affected families has not been studied in Greece.

## Primary Objectives

The unraveling of the biopsychosocial consequences in these parents regarding anxiety and/or depression, the ways of their coping (WOC) to stressful stimuli and their adherence to physicians’ and physiotherapists’ instructions.

## Secondary Objectives

The correlation of these consequences with the disease activity marker (MDVAS), the CHAQ and pain (VAS).

## Materials and methods

76 parents of 69 JIA children (56 mothers: 20 fathers, 14/76 in couples, mean age 39.9 and 42.8 years respectively) were assessed using specific and validated in the Greek population tools. These tools score the anxiety (Spielberger), the depression (Beck), the WOC (Lazarus & Folkman) and the adherence to the health professionals’ instructions.

## Results

The mothers demonstrated high scores regarding anxiety, either as a state (p=0.0001) or a trait (p=0.01) and symptoms of mild depression (10.28±7.60). As of the WOC, the fathers demonstrated lower scores in emphasizing the positive (p=0.004), whereas both parents demonstrated high scores of confrontable coping (p=0.0006 and p=0.02). Parents had a very satisfactory adherence to health professionals’ instructions and a concordance was observed within the couple. No significant correlation was detected between the biopsychosocial consequences and the parents’ assessment of JIA activity and outcome.

## In conclusion

The biopsychosocial consequences of parenting a child with JIA are mild regarding anxiety and depression; the parents are demanding and adhere to health professionals’ instructions. However, these consequences do not correlate with the markers of the JIA course and outcome.

